# Development of behavioral parameters and ERPs in a novel-target visual detection paradigm in children, adolescents and young adults

**DOI:** 10.1186/s12993-015-0067-7

**Published:** 2015-07-04

**Authors:** María Ángeles Rojas-Benjumea, Ana María Sauqué-Poggio, Catarina I. Barriga-Paulino, Elena I. Rodríguez-Martínez, Carlos M. Gómez

**Affiliations:** Human Psychobiology Laboratory, Experimental Psychology Department, University of Seville, Sevilla, Spain

**Keywords:** Frontal Selection Positivity, Selection Negativity, P3a, P3b, Slow Wave, Development, Children

## Abstract

**Background:**

The present study analyzes the development of ERPs related to the process of selecting targets based on their novelty.

**Methods:**

One hundred and sixty-seven subjects from 6 to 26 years old were recorded with 30 electrodes during a visual target novelty paradigm.

**Results:**

Behavioral results showed good performance in children that improved with age: a decrease in RTs and errors and an increase in the d′ sensitivity parameter with age were obtained. In addition, the C response bias parameter evolved from a conservative to a neutral bias with age. Fronto-polar Selection Positivity (FSP) was statistically significant in all the age groups when standards and targets were compared. There was a statistically significant difference in the posterior Selection Negativity (SN) between the target and standard conditions in all age groups. The P3a component obtained was statistically significant in the emergent adult (18–21 years) and young adult (22–26 years) groups. The modulation of the P3b component by novel targets was statistically significant in all the age groups, but it decreased in amplitude with age. Peak latencies of the FSP and P3b components decreased with age.

**Conclusions:**

The results reveal differences in the ERP indexes for the cognitive evaluation of the stimuli presented, depending on the age of the subjects. The ability of the target condition to induce the modulation of the studied components would depend on the posterior-anterior gradient of cortex maturation and on the gradient of maturation of the low to higher order association areas.

## Introduction

The selection of a stimulus as a target is a complex process that involves a certain number of cognitive operations indexed by several Event Related Potential (ERP) components. The present report explores how the different ERPs related to the selection for action of novel stimuli develop with age.

This process has been extensively studied during selection based on a certain feature, color, line orientation, etc. The selection of targets based on non-spatial features, such as color or shape, induces a negative ERP: The so-called *Selection Negativity* (SN), which begins between 140 and 180 ms post-stimulus and persists for another 200 ms or more [1]. SN reflects the selection of visual stimuli at an early level of information processing [2]. It has been proposed that the representation of the selected feature must be active in order to permit the matching between the selected feature and the stimulus containing it. This type of stimulus selection is assumed to be based on a rapid analysis of the physical features of the stimuli that occurs before all the stimulus properties are fully analyzed. However, it is also possible to imagine an experimental paradigm in which the selection would take place by differentiating the presented item from a certain endogenously activated image. This would be, for instance, the case of a child looking for pictures of football players or *pokemons* that are not yet in his/her collection. Although this situation seems to be very common in a child’s life, it has been studied very little. In the experimental paradigm in the present study, novel visuals are chosen as targets to explore the mechanisms involved in the selection of new elements. SN is best observed in difference waves, where the ERP elicited by a stimulus with the unattended feature is subtracted from the ERP elicited by the same stimulus when it has the relevant attended feature. SN latency would indicate the timing of the selection process, while the localization of the SN neural sources would provide information about brain areas involved in the attentional selection of different stimulus features. The timing and brain sources of the SN during attention to color in young adults were elicited during the 160–350 ms interval after stimulus onset and focused on the posterior visual cortex [3]. Van der Stelt et al. [4], in a developmental study using a color selection paradigm, found SN to be an occipito-temporo-parietal distributed negativity in the 150–300 ms latency range. SN was clearly visible for the 19–24 and 16–18 year old subjects, and to a lesser extent for the youngest subjects. This negativity was preceded by Frontal Selection Positivity (FSP) and followed by a P3b that showed decreasing latency with age. In a study comparing ADHD children with controls (between 7 and 13 years old) on an early selective attention task in the visual modality, Jonkman et al. [5] found an occipital selection negativity at Oz electrodes from 200 to 280 ms in both groups. They proposed that the neural sources of the SN of control children and ADHD children were due to a couple of posterior dipoles. These results indicate that selective mechanisms are also operating in children during attentional tasks. However, it is not known whether an SN appears in children when novel and unknown targets are compared with a frequent standard.

Following the SN, the frontal P2 is related to the selection of relevant features and, depending on the experimental paradigm, can lead to target recognition. The frontal P2 component is also related to target processing. This P2 component has also been described as P2a, P2f, the Frontal Polar component (FP), and FSP [6–8], which peaks around 200 ms of the relevant stimuli. This component has been related to the processing of task-relevant stimuli in the transition from the selection of relevant features to the selection of responses [7]. However, given that the P2 component increased in both overt and covert responses, Potts [8] suggested that P2a is related to task-relevant stimuli processing in the stimulus evaluation operation rather than to the selection of motor responses. Regarding the development of P2, an increase in amplitude with age has been shown in the auditory modality [9]. In the visual modality, Van der Stelt et al. [4] did not find amplitude differences among the different age groups, but they found a decrease in latency with age. In the study by Jonkman [5], the FSP of control children presented early sources in medial and posterior areas, and late sources in frontal areas. From here, we would use the FSP terminology for frontal positivity (around 200 ms post-stimulus) when referring to the different wave of ERPs induced by a target minus ERPs induced by a non-target, and P2f and P2p for positive ERPs induced by targets at frontal and posterior sites at around 200 ms, respectively.

When an infrequent stimulus is presented, whether target or non-target, a positive P300 component is induced with a latency of around 300 ms post-stimulus. This component was first observed by Sutton [10] and presents two sub-components: the P3a with fronto-central topography and latency around 250–300 ms, and the P3b, which occurs later in time and is observed in parietal sites. This latter sub-component corresponds to a late positive component elicited around 300 ms after the infrequent stimuli are presented [11–14], and its amplitude is inversely related to the probability of stimulus appearance. Donchin and Coles [15] proposed that P3b would represent a context-updating operation and subsequent memory storage [16]. By contrast, Mangun and Hillyard [17], in a central cue Posner paradigm, showed that adults presented an increased P3b to invalidly cued targets compared to validly cued targets, and they interpreted this increase as a reaction to failed expectancies. Verleger et al. [18] suggested that P3b is related to the neural linkage between stimulus perception and the response to that stimulus. Polich [16] proposed that the P3b component is related to the neuro-inhibition needed to focus attention on the relevant task, facilitating the interference-free action of memory systems [16]. The P300, or in more general terms the Late Positive Component, can be elicited by targets (the so-called target P3), so that a repeated target (target-P3) must be discriminated among distractors, or it can be elicited by rare distractors (novel P3).

P3a, the other sub-component of the P300, is generated as a brain response to stimuli that are novel compared to more frequent stimulation [19, 20]. P3a presents two subcomponents, early and late P3a, respectively [19]. A larger P3a [20, 21] would thus indicate that a stimulus is processed as a novel stimulus that required a shift in attention [16]. The P3a component is also elicited when a switch in the processing rules is needed [22, 23]. It has also been proposed that both the frequency of the presentation of the stimulus and the subjective expectancy of the target are related to the generation of the P3a component [24, 25]. Frontal lobe lesions were found to produce a decrease in the amplitude of P3a. However, P3b recorded in parietal areas is not modified by frontal lesions [26, 27]. The P3a reduction after lesions is not only produced by lateral prefrontal cortex damage, but also by lesions in the orbitofrontal cortex [27]. However, lesions in the temporo-parietal junction reduced the amplitude of both components. Therefore, the P3a and P3b generators are rather distributed, as P3a presents a high dependence on frontal and temporo-parietal junction areas, while P3b generation is not dependent on frontal areas and is distributed in mainly posterior sources [28].

Several studies have analyzed the amplitude and latency of the endogenous P3a and P3b components during childhood development in control subjects in the auditory modality. Using the three-stimulus oddball task (standards, repeated targets and novel distractors), Määttä et al. [29] analyzed children from 8 to 9 years old and adults from 22 to 28 years old. These authors reported a maximum P3a in the frontal areas in children, whereas in adults P3a was highest in central areas. In another study using the oddball task, Fuchigami et al. [30] observed that the latency of both P3a and P3b decreased with age, and that P3a matures earlier than P3b. Oades et al. [9] replicated decreased latency with increasing age for the P3b component. However, other studies using the auditory go-no go task with subjects between 7 and 25 years old reported that, although younger children produced P3b, P3a showed inconsistent patterns. Segalowitz and Davies [31] found that 13 year-old children started to show the standard auditory P3a adult pattern. Gumenyuk et al. [32], using the distraction paradigm with children between 8 and 13 years old, reported the presence of a P3a component in the auditory modality. Wetzel and Scröger [33] found a P3a component to novel sounds in children from 6 to 8 years old. Auditory P3a has been shown to be elicited in children from 2 to 3 years old by stimulation with novel sounds and a variety of different deviant features [34]. Given the immaturity of the frontal cortex in 2–3 year old children, auditory cortex generators have been proposed for this P3a-like component in young children. P3a auditory developmental studies have been reviewed by Wetzel and Schröger [35]. With regard to auditory P3b, the most frequently reported general trend is an increase in amplitude from childhood to adulthood [36].

Only a few studies have analyzed visual modality P3 maturation in children. With a three-stimulus oddball task, Stige et al. [37] analyzed two age groups (6.8–15.8 and 20–88.8 years old), and found that P3a matures earlier than P3b. They showed that the latency of P3a increased with age, and the amplitude decreased with age. The P3b component did not change with latency and showed a non-linear reduction in amplitude with age. Conversely, Courchesne [38] did not find a visual P3a component in children, but he reported longer P3b latencies and amplitudes in children than in adults. Additionally, in this seminal study, he found a frontal negativity at around 410 ms (Nc) and a late fronto-polar positivity around 900 ms (Pc) in children, but they were not present in adults. From these data, the author concluded that these differences are due to different event processing in children, young adults and adults.

In addition to the previously indicated components, a centro-frontally distributed N2b in the 200–450 ms time range and a frontally distributed Late Processing Negativity (LPN), also called Slow Wave (SW), in the range of 300 and 700 ms, have been described as part of feature selection processing and target post-processing, respectively [39].

Although there are some inconsistencies in the reported results on ERP development, particularly in the case of P3a, it is a well-established fact that the maturation of posterior primary sensory cortices precedes the maturation of higher order association cortices, including the dorsolateral prefrontal cortex, inferior parietal and superior temporal gyrus (see review by Giedd et al.,) [40]. The late maturation of frontal ERP components has been proposed for Contingent Negative Variation (CNV) and Error Related Negativity (ERN) [31, 41, 42], for the motor component of CNV [41, 42], for the Lateralized Readiness Potential (LRP) [43] and for the Negative Slow Wave (NSW) [44], a component related to item retention on working memory tasks. All these results suggest that it is possible, in spite of contradictory previous results, that delayed frontal maturation can be expressed as a slower age-related maturation of frontal ERP components.

The present study focuses on analyzing the ERPs related to the target selection process in a novel-target paradigm. This study is motivated by the low number of studies addressing ERP maturation in the visual modality during stimulus selection and by the variability in the results obtained. The main hypothesis is that, in the maturation of the ERP components generated in posterior areas such as the SN, P3b would occur earlier than FSP, P3a and SW, which have critical contributing sources in frontal areas. The present study provides a global landscape of ERPs, indexing the selection process of novel stimuli compared to a repeated standard stimulus, and discerning the role of frontal lobe maturation in this process.

## Methods

### Sample

This study included a sample consisting of 167 human subjects between 6 and 26 years old (4 females and 4 males for each year of age). Twelve subjects were excluded due to excessive EEG artifacts. In the whole sample, 143 were right-handed and 12 left-handed. The left-handed subjects were maintained to increase the generalizability of the results. The group of males consisted of 80 subjects (mean age of 16.14 ± 6.15 SD age), 73 right-handed and 7 left-handed. The female group consisted of 75 subjects (mean age of 16.68 ± 5.88 SD), 70 right-handed and 5 left-handed. Subjects did not report any neurological diseases or psychological impairments. For the behavioral analysis, all the subjects were included to increase the analytic power, although for the ERP analysis some subjects were discarded due to a high number of artifacts in the EEG (see below). Subjects were assigned to five age groups for statistical purposes. Table 1 presents the demographic characteristics of the total and the reduced sample for ERP analysis. All subjects were recruited from middle-class socioeconomic backgrounds. The children had normal academic records, and the young adults were college students. Experiments were conducted with the informed and written consent of each participant (parents/tutors in the case of the children), following the Helsinki protocol. The study was approved by the Ethical Committee of the University of Seville.

**Table 1:** Summary of the demographic variables in the total sample and in the reduced sample after discarding the 12 subjects in which a high number of trials were contaminated by EEG artifacts

Sample	Number	Females %	Males %	Mean of age	Standard deviation of age
Full sample	167	50.90	49.10	15.8	6.06
Group: 6–9 years	33	51.52	48.48	7.48	1.14
Group: 10–13 years	32	53.13	46.87	11.47	1.16
Group: 14–17 years	32	50.00	50.00	15.5	1.14
Group: 18–21 years	33	48.49	51.51	19.55	1.15
Group: 22–26 years	37	51.35	48.65	24.02	1.44
Reduced sample	155	48.38	51.62	16.4	5.99
Group: 6–9 years	25	44	56	7.36	1.11
Group: 10–13 years	28	46.43	53.57	11.53	1.2
Group: 14–17 years	32	50	50	15.5	1.14
Group: 18–21 years	31	48.39	51.61	19.54	1.18
Group: 22–26 years	39	51.28	48.72	24.05	1.41

### Experimental paradigm

**Fig. 1:**
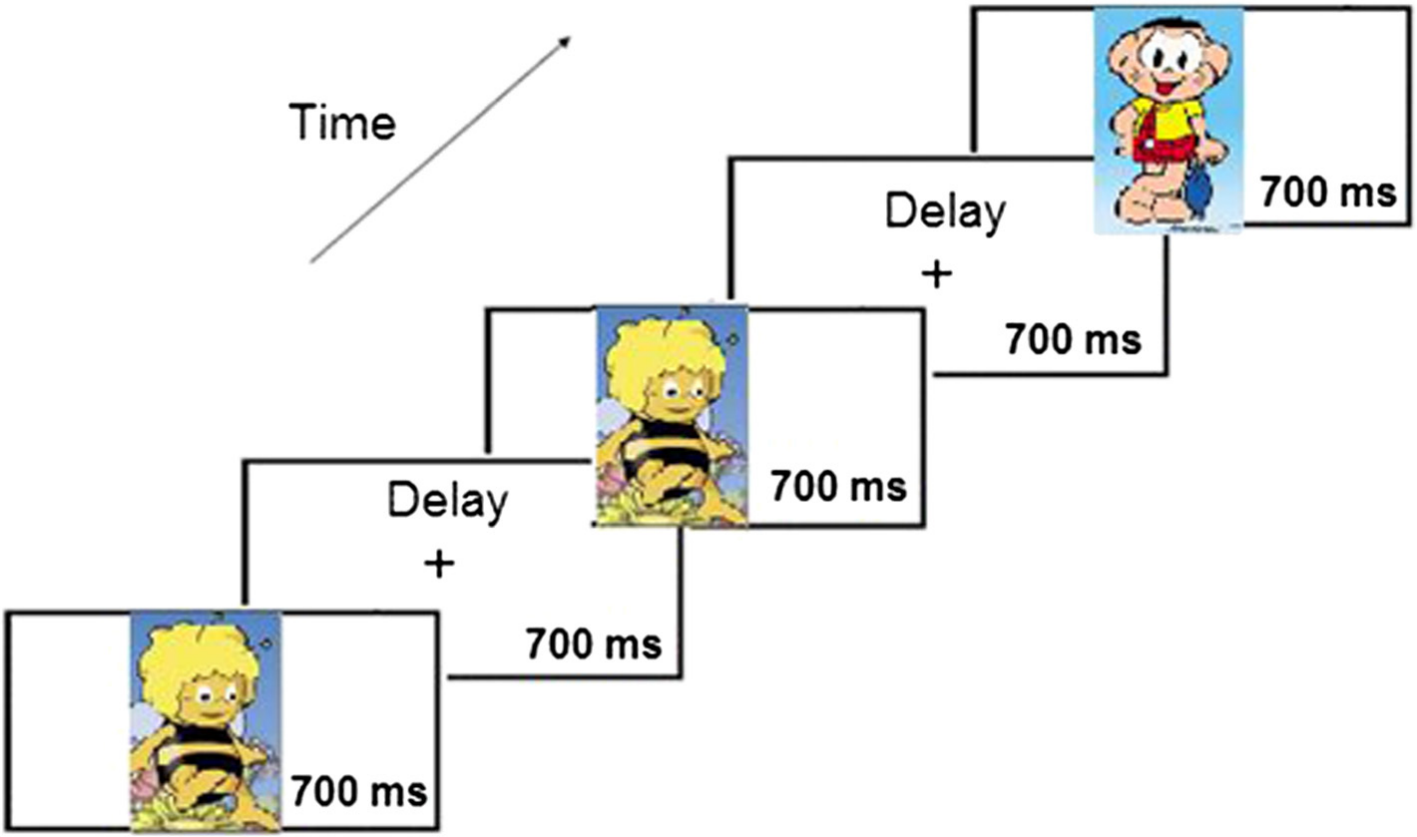
An example of a trial of the Oddball task. Presentation of a sequence of cartoons where the bee was the frequent standard stimulus and other cartoons were the infrequent novel target stimuli. The subject had to respond to the novel stimuli. See the details in the text

Visual stimuli were cartoons. The size of all the stimuli was adapted in Picassa to equal dimensions of 142 × 228 pixels. The stimulus presentation program used was E-Prime version 2.0, and a SRBOX Cedrus was used to record the subjects’ responses. The novel-target visual paradigm was composed of a total of 120 trials; 25 % of them were novel stimuli, and 75 % were the same standard stimuli. The stimuli were presented at the center of the screen for 700 ms with an ISI of 700 ms, covering a visual angle of 4.56° and situated 2.28° eccentrically in the horizontal meridian. The subjects were told to consider novel stimuli as targets. Subjects were instructed to press the button only when a novel stimulus appeared. The response window was 1400 ms. There was only one block (90 frequent and 30 infrequent stimuli), and the order of stimuli presentation was random. Figure 1 shows an example of a task trial.

The behavioral variables obtained were RTs, errors and parameters derived from signal detection theory (see below). Means, standard deviations and variation coefficients for the RTs were computed for individual subjects, as well as the means for different types of errors: False alarms (responses to non-target stimuli), omissions, anticipations (responses lower than 200 ms), total errors, d′ and C parameters. To compute the d′ and C parameters, the following equations were used [45].

1 $$ \mathrm{d}^{\prime }={\uptheta}^{-1}\left(\mathrm{H}\right)-{\uptheta}^{-1}\left(\mathrm{F}\right) $$

θ^−1^(H): Z values of the proportion of hits
θ^−1^(F); Z values of the proportion of false alarms

High positive values are related to high sensitivity, while zero values indicate low sensitivity, and negative values would indicate poor comprehension of the instructions, with the subject responding to standard rather than target stimuli.

$$ \mathrm{C}=\left({\uptheta}^{-1}\left(\mathrm{H}\right)-{\uptheta}^{-1}\left(\mathrm{F}\right)\right)/2 $$

C positive values are obtained when subjects show a conservative response bias, C negative values are considered to be related to a liberal response bias, and C values near zero indicate a neutral tendency to respond (not biased).

### EEG recording

Subjects were recorded at different times of the day between 12 AM and 8 PM. No information about previous sleep was required. Recordings were obtained from an average reference of 32 scalp sites following the international system (Fp1, Fpz, Fp2, F7, F3, Fz, F4, F8, FC5, FC1, FC2, FC6, M1, T7, C3, Cz, C4, T8, M2, CP5, CP1, CP2, CP6, P7, P3, Pz, P4, P8, POz, O1, Oz, O2), using tin electrodes mounted on an electrode cap (ELECTROCAP). Eye movements were recorded by two electrodes at the outer canthus of each eye for horizontal movements, and by electrodes placed above and below the left eye for vertical movements. All the scalp electrodes were re-referenced offline to the mastoid average (M1 + M2)/2. Impedance was maintained below 10 kΏ. Data were recorded in direct current (DC) mode at 512 Hz, with a 20,000 amplification gain using a commercial Analogy Digital (AD) acquisition and analysis board (ANT). Data were not filtered during registration. We asked subjects to stay calm and look at the screen while blinking as little as possible.

EEG recordings were analyzed with the EEGLAB [46] software package. Before applying Independent Components Analysis, the data were offline filtered by means of a high (1 Hz cut-off frequency) and low pass (25 Hz cut-off frequency) Finite Impulse Response filter implemented in EEGLAB (pop_ eegfilter function). The cut-off frequencies were established following the clinical criteria for ERPs [47]. To eliminate AC power line interference and blink artifacts in the EEG, an Independent Components Analysis [48] was performed. Criteria for determining these artifact components were their scalp map distribution, time course and spectral power. The eye blink artifact component showed a frontal location, coincided with blinking in the recording of eye movements, and showed low frequency in the power spectrum. Electromyography artifacts were located in temporal electrodes and presented a high frequency burst. These components were discarded, and the EEG signal was reconstructed. Epochs of 1600 ms were obtained: from −200 ms pre-stimulus to 1400 ms post-stimulus. Baseline was in the −50 to 0 ms period in the epoch. ERPs were obtained by averaging time, locked to the stimulus. Twelve of the 167 subjects recorded were excluded from the analysis due to a high number of ocular blinks, EMG, and trend-derived contaminations in the EEG.

Artifact corrected recordings were averaged off-line using a rejection protocol based on voltage amplitude. The recorded voltages that exceeded ±100 μV in the recordings of subjects from 16 years old on, and ±150 μV in the recordings of subjects up to 15 years old in any channel, were rejected for further analysis in order to eliminate any extra-cerebral contamination. The application of distinct voltage values was due to the known difference in the spectral power of children’s and adults’ recordings because children present higher spectral power than adults do [49–51]. If we had applied ±100 μV to all subjects, which is usually the standard value applied for artifact rejection, many non-contaminated trials of the children’s recordings would have been eliminated from the electrophysiological data. The ERPs obtained from children presented a similar noise level and baseline to those of adults (see Figs. 3 and 4), indicating that the procedure for selecting different voltage windows for artifact rejection for different ages was appropriate and did not distort the results of the inter-group and intra-group comparisons. Additionally, as the main statistical comparison would be between the amplitudes of the target and standard conditions within each group, and both conditions would be processed with the same rejection limits, the comparisons should not be affected. The total number of trials in each condition and age group is displayed in Table 2.

**Table 2:** Number of accepted trials in the different age groups

Age groups	Average		Minimum range		Maximum range	
Years	Std	T	Std	T	Std	T
6–9	65.24	22.08	39.00	13.00	85.00	30.00
10–13	66.86	23.82	30.00	11.00	87.00	30.00
14–17	76.75	24.10	51.00	10.00	90.00	30.00
18–21	78.65	26.84	47.00	12.00	90.00	30.00
22–26	80.21	27.33	52.00	20.00	89.00	30.00

*T* target condition, *Std.* Standard condition

The algebraically-linked mastoids were computed off-line and used as a reference for analytical purposes. ERPs were obtained for each subject by averaging the EEG, using the switching-on of the stimuli as a trigger. Target and standard stimuli were averaged separately.

### Statistical analysis

#### Behavioral analysis

The developmental trajectories of the different behavioral measurements (RTs, standard deviations of RTs, variation coefficients of RTs, false alarms, omissions, anticipations, total errors, d′ and C parameters) were established by regressing them with age (in days). Linear and inverse models were tested.

#### ERP analysis

The ERPs were obtained independently in the different age groups (Children: 6–9 years old; Pre-adolescents: 10–13 years old; Adolescents: 14–17 years old, Emergent adults: 18–21 years old and Young adults: 22–26 years old).

In the present study, the primary interests were the SN, the FSP, and the target’s modulatory effect on the P3a P3b and SW components, which increased their amplitude in the target stimuli compared to the standard stimuli. Therefore, to select time windows, the difference wave was obtained by subtracting ERPs induced by standards from ERPs induced by targets, and time windows in young adults were chosen for FSP and SN in 200–280 ms; for P3a in 340–380 ms, and for P3b and SW in 380–500 ms. The subtraction is performed for representation purposes. However, the statistics are based on the comparison of the ERPs in the standard vs. the target conditions; for instance, to find out whether there was a significant FSP, a significant difference between P2 in the standard and target conditions must be obtained (the same is true for SN, SW, P3a and P3b). In the children’s group, and after averaging and analyzing the ERPs and topographies, it was evident that the FSP component was delayed compared to the other age groups; for this reason, the chosen latency for FSP in children was 280–360 ms. Similarly, for the pre-adolescent group, a delayed P3a component was evident; therefore, the P3a time window for the children’s and pre-adolescent groups was 490–530 ms. For the same reason, the selected time window for analyzing P3b in children and pre-adolescents was delayed to 530–700 ms. The N2b component was not evident in the recordings and, therefore, was not analyzed.

Difference-wave topographical maps were obtained for the selected time windows. Taking into account the extensive literature previously cited and the topography of the recordings in the five age groups, the following electrodes were chosen for further ERP analysis: FP1, FPz and FP2 for the FSP component; P3, Pz, P4, POz, O1, Oz and O2 for the SN component; F3, Fz F4, FC1 and FC2 for the P3a component; P3, Pz, P4, POz, O1, Oz and O2 for the P3b component; and FP1, FPz and FP2 for the SW component.

#### ERP statistical analysis

A mixed-model ANOVA was computed independently for each component. The between-subjects factor was the age group (five age groups). The within-subjects factors were the experimental condition (target and standard) and the electrodes (depending on the component). When an interaction between the effect of the condition and the age group was obtained, the Bonferroni correction for multiple comparison t-tests was computed between ERP mean voltages in the standard and target conditions for each age group. These comparisons would reveal whether ERPs in the target condition were statistically different from ERPs elicited by standard stimuli in the different age groups. Other comparisons (changes in amplitude with age and electrodes) will be reported, but not explored with post-hoc tests, given that they do not correspond to the primary interest of the present report.

The latency of the different components in the difference waves was estimated by the min/max functions in Matlab in selected electrodes for each component. Broader time windows than those used to compute amplitudes were tested to find component peaks: FSP (Fpz: 200–360 ms), SN (POz: 260–360 ms)), P3a (Fz: 340–530 ms), P3b (Pz: 380–700 ms) and SW (FPz: 380–700 ms).

Spearman correlations were computed for the different ERP components obtained in the difference waves and the nine behavioral measures included in the present study.

## Results

### Behavioral measures

**Fig. 2:**
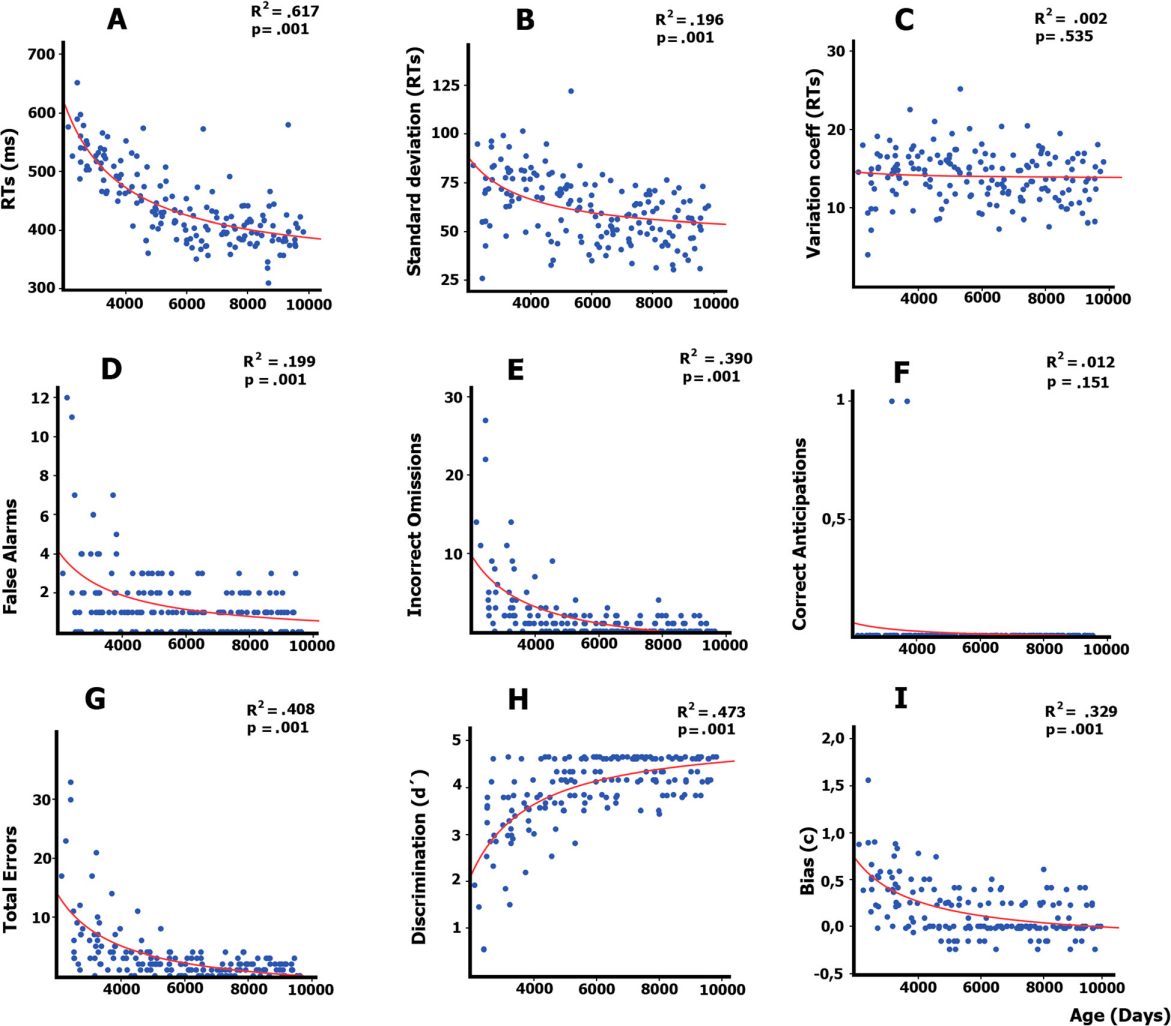
Inverse model regressions between the age expressed in days and the behavioral parameters: RTs (**a**), the standard deviations of RTs (**b**), the Coefficient of Variation (**c**), false alarms to standard stimuli (**d**); omissions to targets (**e**) anticipations to targets (**f**); total errors (**g**), d′ sensitivity parameter (**h**), and the response bias parameter (C) (**i**). Errors were expressed in percentage of errors for each error category

The linear and inverse regressions between age and the different behavioral parameters of RTs, errors, and signal detection theory parameters were obtained (Fig. 2). Linear and inverse models were tested, and the inverse models explained more variance than the linear models, estimated with R^2^ values. The statistical analysis of the regression between the mean RTs vs. age (in days) (Fig. 2a), and of the standard deviations of RTs vs. age (Fig. 2b), showed a significant inverse relationship between the two variables. The Coefficient of Variation did not show a statistically significant relationship with age (Fig. 2c). Most errors were due to false alarms to standard stimuli (Fig. 2d) and omissions to targets (Fig. 2e). Both were inversely related to age. The less frequent anticipation errors were also inversely related to age (Fig. 2f). Total errors also presented a significant inverse relationship with age (Fig. 2g). The d′ parameter increased with age (Fig. 2h), and the response bias C parameter decreased with age (Fig. 2i), indicating an increase in perceptual discrimination and a transition from a conservative to a neutral response bias with age, respectively. Table 3 shows the values for the behavioral parameters in each age group.

**Table 3:** Mean an standard deviation of the nine behavioral parameters in the five age groups

Age group	RTs		SD RTs		CV		Number of false alarms		Number of omissions	
Years	Mean	SD	Mean	SD	Mean	SD	Mean	SD	Mean	SD
6–9	537.38	41.09	72.41	18.00	13.9	3.2	2.55	2.83	5.76	6.12
10–13	493.44	39.90	74.95	12.73	15.0	3.4	1.87	1.52	1.80	2.07
14–17	427.92	34.58	63.92	17.05	14.2	3.5	1.18	1.02	0.71	1.05
18–21	410.53	44.80	52.59	11.44	13.9	2.8	0.75	0.90	0.57	0.93
22–26	399.37	39.24	54.80	12.52	13.2	3.0	0.91	0.86	0.48	0.73
Age group	Number of anticipations		Number of total errors				d′		C	
Years	Mean	SD	Mean		SD		Mean	SD	Mean	SD
6–9	0.02	0.17	8.35		7.91		2.90	1.07	0.50	0.34
10–13	0.03	0.17	3.70		2.92		3.62	0.62	0.30	0.28
14–17	0.00	0.00	1.90		1.57		4.08	0.46	0.06	0.20
18–21	0.00	0.00	1.33		1.31		4.32	0.38	0.08	0.17
22–26	0.00	0.00	1.40		0.95		4.33	0.34	0.05	0.19

*RTs* Reaction Times, *SD* Standard deviation, *CV* Coefficient of Variation, *d′* Sensitivity Index, *C* Response Bias Index

### Event-Related Potentials (ERPs)

**Fig. 3:**
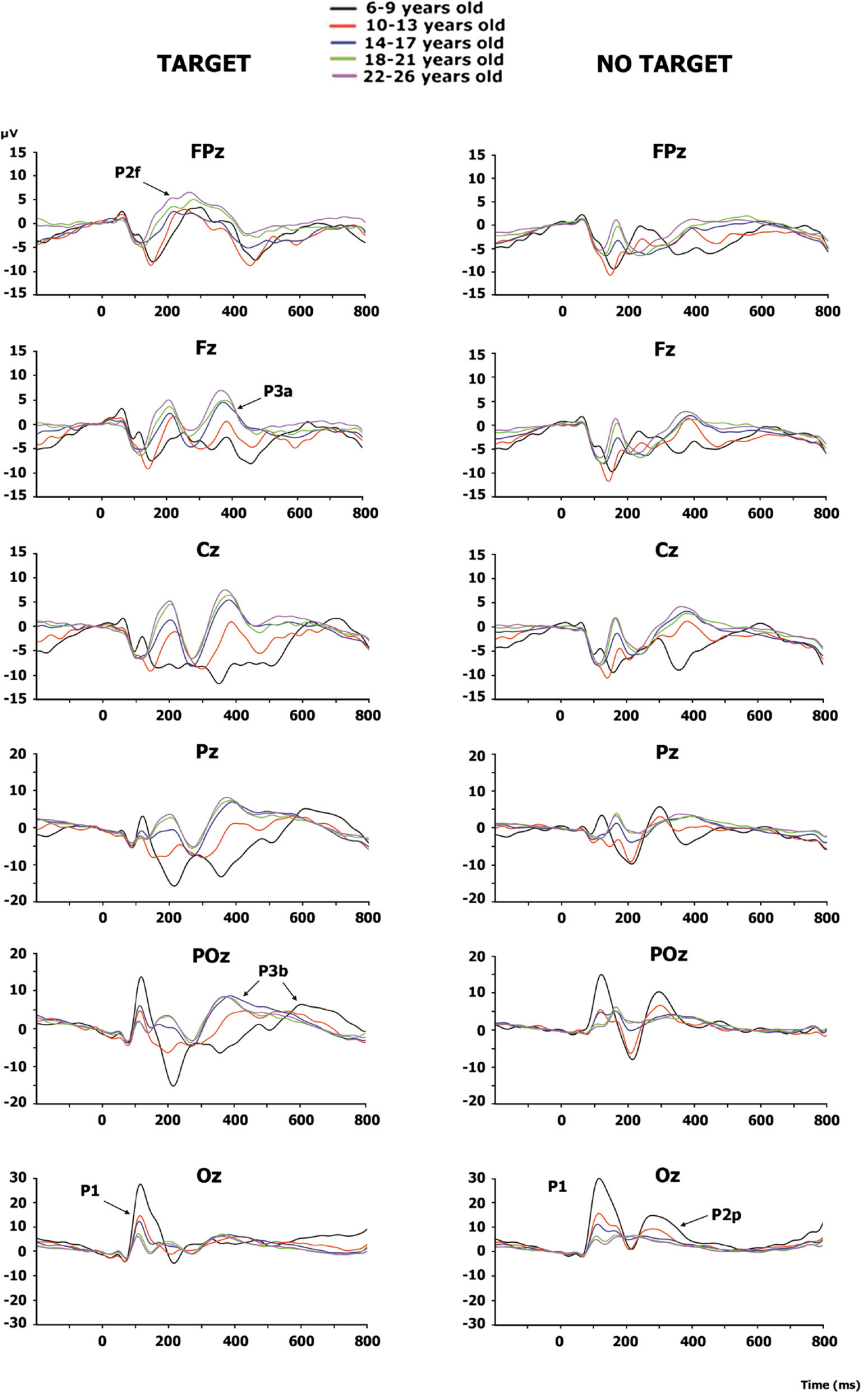
ERPs for midline electrodes in the two studied conditions: target and standard. The amplitudes elicited by the target stimuli were higher than the amplitudes elicited by the standard stimuli in most components and age groups. In addition, the different morphologies of ERPs in the two conditions can be observed. The components P2f (P2 frontal), P2p (P2 posterior), P3a and P3b are indicated by *arrows*

**Fig. 4:**
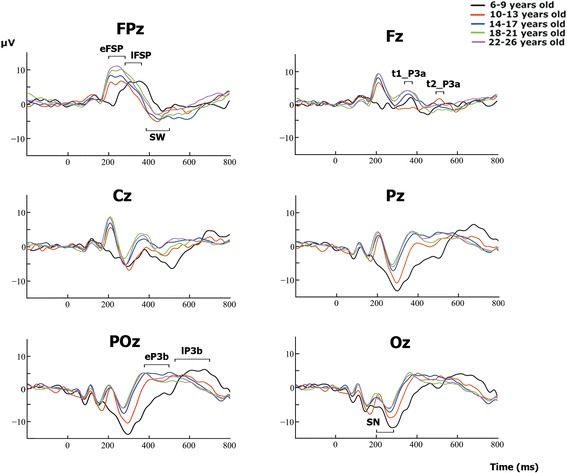
Difference waves obtained by subtracting the ERPs in the standard condition from the ERPs in the target condition. The differences between age groups seem to be due to different latencies rather than to different morphologies. The *horizontal bars* indicate the time windows used for statistical analysis. Two different time windows marked for a component indicate that for the statistics, the early window is used for the older subjects and late latencies for younger subjects (see details in the Methods and Results sections). *eFSP* early Frontal Selection Positivity, *lFSP* late Frontal Selection Positivity, *SN* Selection Negativity, *t1_P3a* early latency P3a, *t2_P3a* Late latency P3a, *eP3b* early P3b, *lP3b* late P3b, *SW* Slow Wave

**Fig. 5:**
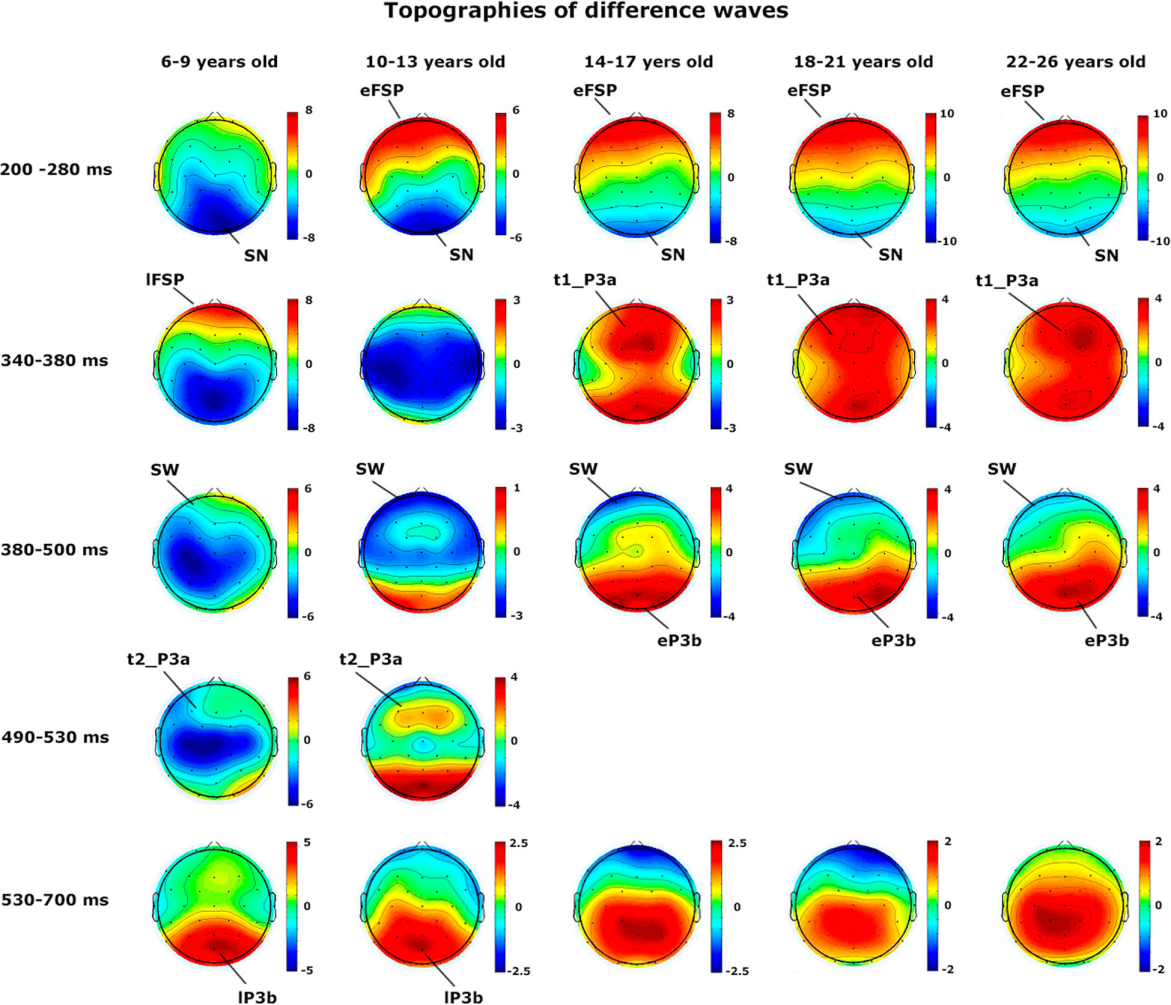
Topographies of the different components of the difference wave in different latencies for the different age groups. *eFSP* early Frontal Selection Positivity, *lFSP* late Frontal Selection Positivity, *SN* Selection Negativity, *t1_P3a* early latency P3a, *t2_P3a* Late latency P3a, *eP3b* early P3b, *lP3b* late P3b, *SW* Slow Wave

**Fig. 6:**
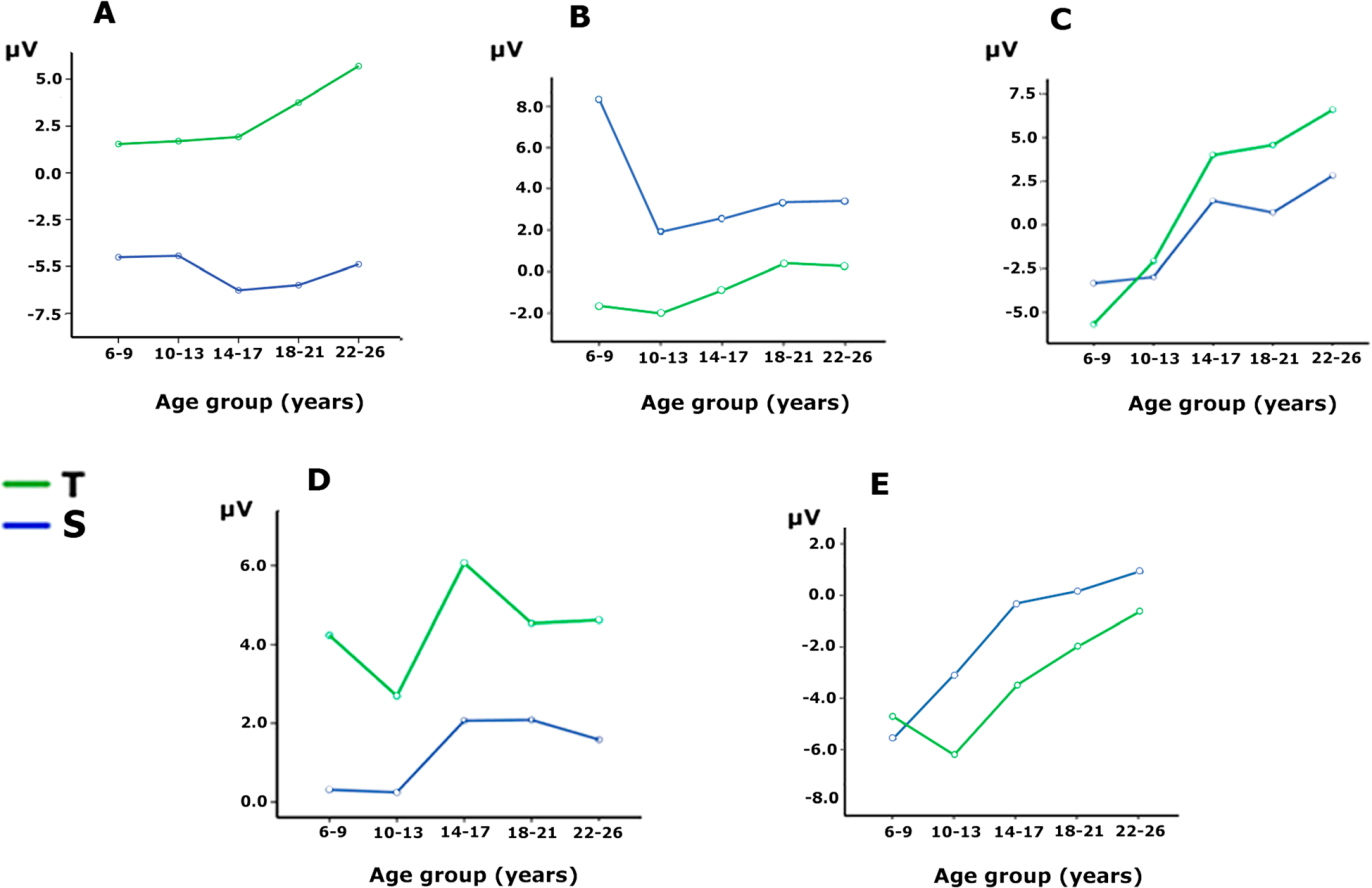
Mean amplitude values of the ERPs in target and standard conditions at the latencies of the P2f, P2p, P3a, P3b and SW components (**a**, **b**, **c**, **d** and **e**, respectively). FSP and SN must be interpreted as the difference waves of the target minus standard conditions in (**a**) and (**b**). The represented amplitudes correspond to the average voltage amplitude in the selected electrodes for each component (see the Methods section). *T* Target, *S* Standard

Figure 3 shows the ERPs for mid-line electrodes in the two studied conditions: target and standard stimuli. The amplitudes for target stimuli were higher than those for standard stimuli. Children and pre-adolescents presented a different morphology of ERPs compared to adults. The difference wave obtained by subtracting the ERPs of standard stimuli from the ERPs of target stimuli is presented in Fig. 4. The morphology of the difference wave suggests that differences between age groups are due to different latencies rather than to different morphologies, given that the same components appeared in all the age groups (except P3a in children), although with later latencies in the two younger groups. Figure 5 presents the topographies of the different components obtained from the difference wave for the different age groups. Figure 6 shows the mean ERP values in the target and standard conditions in the latencies corresponding to the SN, FSP, P3a, P3b and SW components.

#### P2 component: frontal selection positivity

**Fig. 7:**
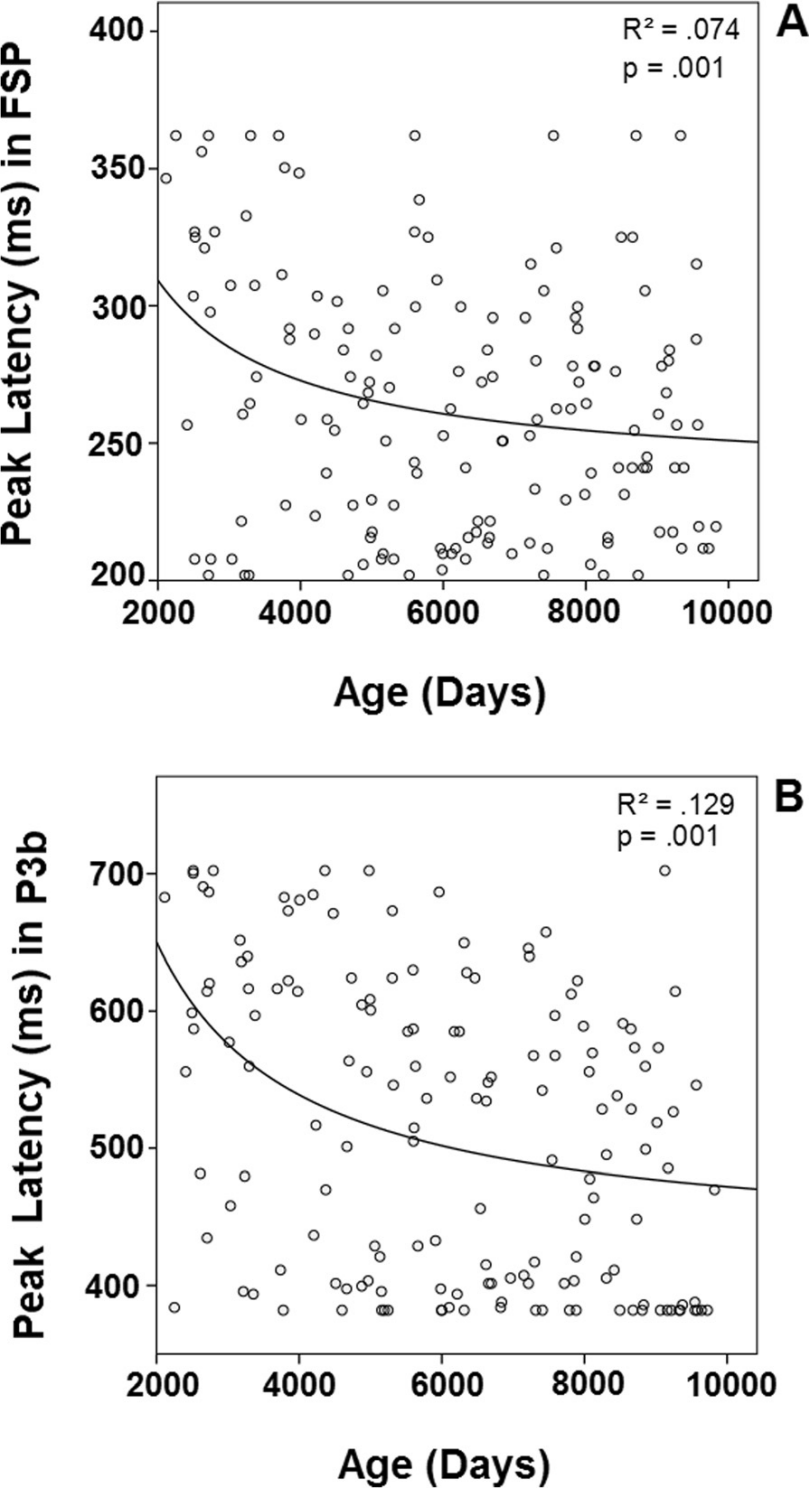
Inverse regression between the peak latency of the ERP components FSP (**a**) and P3b (**b**), with the age expressed in days

The P2 component was clearly recorded in the target condition in anterior electrodes (P2a in Fig. 3), but it was not clearly defined in the standard condition. The difference wave was obtained to observe the FSP. Figure 4 in FPz shows the presence of a delayed FSP in the young children’s group compared to the other age groups. Figure 5 shows the fronto-polar distribution of the FSP, and at the latency of the adult P3a, a FSP-like frontopolar topography appears in children. In fact, in the time window for the adult P3a, a frontopolar positivity can be appreciated in the young children’s group, reinforcing the idea that FSP is delayed in time in children compared to adults. For this reason, FSP has been marked as eFSP (early FSP: 200–280 ms) for the pre-adolescent to adult groups and as late lFSP (late FSP: 280–360 ms) for the children’s group. In fact, the peak latency of FSP showed an increase with age (*p* < 0.001, R^2^ = 0.074, Fig. 7a). Therefore, different time windows were used to compute the statistics for the P2 component in children compared to the other groups.

There were no statistically significant group effects (Fig. 6a). The ANOVA showed that the effects of the electrodes (F [1.85, 278.16] = 18.09, *p* < 0.001) and condition were statistically significant (F [4, 150] = 175.35, *p* < 0.001), the latter due to an increased P2 amplitude in the target condition compared to the non-target condition, corresponding to the presence of an FSP. The interaction between the effects of the age group and the condition was not statistically significant.

#### SN component

The SN component appeared in posterior electrodes in the difference wave (Fig. 4) at the latency of the posterior P2 (P2p, in Fig. 3). The difference wave in the POz and Oz electrodes, displayed in Fig. 4, shows the SN amplitude in children and pre-adolescents compared to older adults. Figure 5 shows the posterior topography of the SN in all age groups. There was no peak latency change with age for the SN component.

The ANOVA showed a statistically significant effect of the age group (F [1, 150] = 3,61, *p* < 0.008) (Fig. 6b). The ANOVA showed that the effects of the electrodes (F [2.11, 317.74] = 324,67, *p* < 0.001) and the condition were statistically significant (F [4, 150] = 74,1, *p* < 0.001), the latter due to an increased amplitude of negativity in the target condition compared to the non-target condition, generating the SN. The interaction between the effects of the age group and the condition was not statistically significant. The SN was not followed by a fronto-central N2b, but it was continued by a fronto-central P3a, probably due to the novel features of the target stimuli.

#### P3a component

The difference wave in the P3a time window showed a fronto-central topography from the adolescent to the adult groups (marked as t1_P3a in Fig. 4). The term early P3a has not been used, in order to avoid confusion with the early P3a sub-component described by Escera et al. [19]). However, at the latency of P3a in adults, the topography in children is similar to the FSP component in adults (Fig. 5). Pre-adolescents showed a delayed P3a (marked as t2_P3a in Fig. 4 to avoid confusion with the late P3a sub-component). Furthermore, at the latency of early P3b, pre-adolescents showed a frontal positivity that can be interpreted as a delayed P3a compared to older groups (Fig. 5). At the time window of the adult P3a, children did not show P3a-like topographies. Therefore, as indicated in the method section, different latencies (t1_P3a: 340–380 ms, t2_P3a: 490–530 ms) were used in children and pre-adolescents, compared to adolescents and adults, for the statistical analysis of P3a. There were no signs of early or late P3a sub-components. However, there was no peak latency change with age for the P3a component.

The ANOVA showed a group effect (F [4, 150] = 23.0, *p* < 0.001) (Fig. 6c). The ANOVA showed that the effects of the electrodes (F [2.62, 394.29] = 33.5, *p* < 0.001) and condition were statistically significant (F [4, 150] = 13.686, *p* < 0.001), the latter due to an increased P3a amplitude in the target condition compared to the standard condition (Fig. 6c). The interaction between the effects of the age group and the conditions was statistically significant (F [1, 4] = 4.22, *p* < 0.003). Bonferroni corrected *t*-test mean comparisons revealed that the P3a in the target condition showed statistically significantly differences from the standard condition only in the young adult (*p* < 0.005) and adult (*p* < 0.005) groups. If Bonferroni corrections are not applied, the adolescent group was also significant (*p* < 0.019).

#### P3b component

The P3b component appeared in posterior electrodes (Fig. 3). Children and pre-adolescents presented a delayed P3b compared to the older groups. The difference wave in the POz and Pz electrodes, displayed in Fig. 4, shows the increased P3b amplitude in the target condition compared to the standard condition, and delayed P3b modulation by condition in the children and pre-adolescent groups. Figure 5 shows the posterior topography of the P3b, and that the posterior topography in children and pre-adolescents is not fully developed until the late P3b latency. In fact, the peak latency of P3b showed an increase with age (*p* < 0.001, R^2^ = 0.129, Fig.7b). Therefore, as indicated in the method section, different latencies were used in children and pre-adolescents, compared to adolescents and adults, for statistical analysis of the P3b (eP3b: 380–500 ms, lP3b:530–700 ms).

The ANOVA showed a group effect (F [4, 150] = 7.42, *p* < 0.001) (Fig. 6d). The ANOVA showed that the effects of the electrodes (F [1.99, 299.11] = 24.72, *p* < 0.001) and the condition were statistically significant (F [1, 150] = 114.038, *p* < 0.001), the latter due to an increased P3b amplitude in the target condition compared to the non-target condition. However, the interaction between the effects of the age group and the condition was not statistically significant, indicating a similar modulation elicited by the target condition with age (Fig. 6d).

**Fig. 8:**
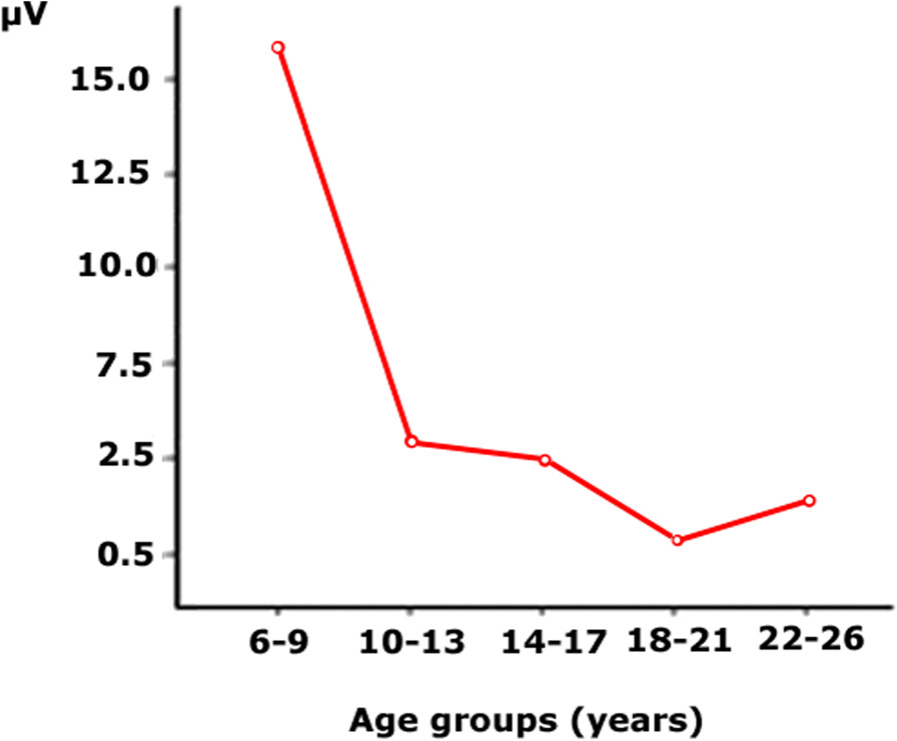
Mean voltage of the peak-to-peak amplitude of the increase in the P3b modulation induced by the target condition. Note the higher P3b in the children’s group (group of 6–9 years) compared to the other age groups

An additional effort was made to demonstrate that there was an increase in the amplitude of the P3b modulation with age if the peak-to-peak amplitude of the P3b modulation was taken into account. For this purpose, the SN (computed from the difference wave of P2p) in the Pz electrode (see Fig. 4) was subtracted from the P3b modulation by the target in the same electrode (also computed from the difference wave). The inter-group ANOVA of this difference showed an age group effect (F [4,150] = 12.58, *p* < 0.001) (Fig. 8), due to the higher amplitude of the peak-to-peak amplitude of P3b in children compared to the other age groups. The Bonferroni corrected comparison showed that the children’s peak-to-peak amplitude was significantly different from that of the other four age groups (*p* < 0.009). No other comparisons were statistically significant.

#### SW component

The SW component appeared in anterior electrodes and presented a higher amplitude in the target condition compared to the standard condition (Fig. 3). The difference wave in the FPz electrode, displayed in Fig. 4, shows the increased negative amplitude of the target condition compared to the standard condition. There was no peak latency change with age in the SW component. Figure 5 shows the anterior topography of the SW. The ANOVA showed a group effect (F [4, 150] = 11.01 *p* < 0.001) (Fig. 6e). The effect of the electrode factor was not significant. The ANOVA showed that the effect of the condition was statistically significant (F [1, 150] = 12.1, *p* < 0.001), due to an increased negative amplitude of the SW in the target condition compared to the non-target condition. However, the interaction between the effects of the age group and condition was not statistically significant (Fig. 6e).

### Correlation between behavioral measurements and ERPs

The different behavioral measurements (presented in Fig. 2) were Spearman correlated with the voltage amplitude and latencies in representative electrodes for the different analyzed ERPs: SN in the Oz electrode, FSP in FPz, P3a in Fz, P3b in Pz and SW in FPz. Because each latency was correlated with nine variables, the Bonferroni correction for multiple comparisons was applied. The mean RTs were statistically significant when correlated with P3b amplitude (Rho = 0.252, *p* < 0.018). The mean RTs were statistically significant when correlated with FSP latency (Rho = 0.219, *p* < 0.05), RTs vs. P3b latency (Rho = 0.353, *p* < 0.009) and SD vs. P3b latency (Rho = 0.225, *p* < 0.048). All of these correlations were positive, and no other correlations were statistically significant.

## Discussion

The present study examined the development of ERPs related to the target selection process in a novel-target paradigm. The present results indicated that similar components to those modulated during the selection of attended features in children [4, 5] were modulated during the selection of visual stimuli considered as targets due to their novel nature, rather than to the presence of a certain feature in the attended channel. The present report focuses on a very ecological process, as it corresponds to selecting visual objects based on their novelty.

The proposed central hypothesis was that, in the maturation of the ERP components generated in posterior areas such as the SN, P3b would occur earlier than FSP, P3a and SW, which have critical contributing sources in frontal areas. This hypothesis was only confirmed by the amplitude parameter for P3a and by the latency parameter in the FSP component. The posterior SN component was already mature in children. Additionally, the P3b component showed a delayed maturation in both the latency and amplitude parameters. These results suggest that not only does an anterior-posterior gradient of maturation occur in ERPs, as indexed by FSP and P3a, but a lower to higher order association area gradient also appears during development, as indexed by P3b.

The behavioral measures showed the classic improvement in performance with age in terms of reductions in mean RTs, RT variability, false alarms, omissions, anticipations and parameter d′ sensitivity. In the age range considered, from 6 to 26 years old, there was a statistically significant inverse relationship with age, indicating a decrease in all these behavioral parameters. Moreover, regarding the response bias parameter (C parameter), there was an inverse relationship with age, indicating that the response bias evolves from a conservative attitude (positive values) to a neutral response bias (values near zero).

The RT reduction with age has been described in visual oddball paradigms [38, 52], which also reported that the most common types of errors in children were omissions [52]. During childhood, as in many other cognitive functions, attention follows a certain developmental trajectory in which RTs and errors decrease with age [53]. This inverse relationship between age and RTs has been extensively obtained in different types of RT experiments [54], and it is probably related to a general factor of psychophysiological maturation. This reduction in mean RTs was accompanied by a reduction with age in RT variability, as obtained in other studies [55, 56]. In the present study, the most frequent types of errors were omissions, followed by false alarms, with a very low number of anticipations. The consideration of omissions as the most frequent error in children was also found in other studies using different paradigms [4, 44, 52, 57–59]. The developmental trajectory showing a decrease in omissions is possibly related to the transition from a conservative to a neutral response bias (C parameter), and it suggests that children present a cautious strategy in responding, as previously suggested on visual search tasks [59], although higher distractibility in children, which would make them more prone to not responding, cannot be ruled out. However, Oades et al. [9], using an auditory oddball task, did not show statistically significant differences between children and young adults in the beta response bias parameter. The conservative response bias in children in the present study is probably due to the complexity of the visual target stimuli, which fostered a cautious strategy in responding. The d′ parameter indexed an increase in the ability to discriminate targets with age, a result also obtained by Oades et al. [9], which corresponds to a well-established trend in child maturation [60].

All the age groups presented a significant, positive fronto-polar component, labeled as FSP in the difference wave (ERPs in target condition minus ERPs in standard condition). The peak latency correlation with age confirmed the delayed build-up of the FSP component in children. Van der Stelt et al. [4] obtained an FSP computed from the difference wave obtained by subtracting the ERPs in the irrelevant color non-target condition from those of the relevant color in the non-target condition. This FSP, extracted as the difference wave in the P2 range, was interpreted as being related to the attentional effort in selecting a relevant color. Similar FSP topographies and latencies are obtained in the present experiment, suggesting that they represent homologous components, and that the present FSP is related to the attentional effort made to discriminate targets from standards. The FSP amplitude did not decrease with age in the Van der Stelt study [4], a result that is confirmed in the present report. The FSP, simultaneous to SN, is related to the selection of relevant features, which, depending on the experimental paradigm, can lead to target recognition [3]. The frontal P2 component is also related to target processing. This component has been related to the process of task-relevant stimuli associated with the selection of responses [7]. However, Potts [8] proposed that P2 is related to the stimulus evaluation operation rather than to the selection of motor responses. An increase in the P2 component with age in the auditory modality has been demonstrated [9], but latency decreases and no amplitude differences have been obtained in the FSP component in the visual modality when age increases [4]. Both results have been confirmed in the present report. The presence of a statistically significant FSP in children in the present study suggests that, in children, this component, which has frontal sources [3–5], is important for novel stimuli and/or associated response selection in children.

Simultaneously with the frontally distributed FSP, an SN was recorded in posterior sites in all the age groups. The SN component is related to the selection of certain non-spatial attended features, such as color, shape, orientation, etc. [3]. However, the negation of the features of the standard was the selection criterion in the present study. The presence of an SN in all the age groups suggests that a similar mechanism to the selection of attended features, the SN, occurs when the negation of certain features is the criterion for target selection. Therefore, it is possible to suggest that a relatively similar mechanism to SN is present in children as a selective mechanism. This result supports previous studies showing the presence of SN in children [4, 5]. These authors, in a color and shape selection paradigm, found SN to be an occipito-temporo-parietally distributed negativity in the 150–300 ms latency range. However, an SN was more clearly visible in the 19–24 and 16–18 year old subjects, and less visible in most of the younger subjects in the Van der Stelt [4] study, in contrast to the present results and to Jonkman et al. [5], who also succeeded in finding an SN in children. The neural sources of the SN component are located in the temporal cortex, corresponding to their role in selecting visual features [3, 5, 61]. The posterior origin of SN would justify the presence of SN in children and pre-adolescents.

Following the FSP and SN, a P3a component was recorded in fronto-central sites. This component was only statistically significant in the emergent and young adult groups. The present results extend previous studies indicating that the visual P3a component would show slow maturation [38, 42], and that it would only be clearly established in young adults. It is difficult to compare the different reported experiments, given the differences in age, sample size, stimuli and paradigms. However, the present results and those obtained by Courchesne [38] and Flores et al. [42] tend to suggest a protracted maturation of the visual P3a component, probably due to the slow maturation of frontal cortical networks. In the Courchesne study [38], a frontal negative component appeared in the P3a component location and latency: the Nc component. Our results and Courchesne’s [38] indicate a lack of clear P3a in the visual modality in children, but Stige et al. [37] found that P3a matures earlier than P3b, showing that P3a latency increased with age, and amplitude decreased with age, throughout the life span. However, small changes appeared when children and young adults were compared, and no Nc component was evident. The present results focus on the novelty and nature of the target stimuli, which can explain the differences with the Stige et al. [37] results. On the other hand, the scarce and diverse results on the visual P3a show the need for further research on this topic. The situation becomes more complicated if results on the P3a in the auditory modality are considered. Results on auditory P3a [9, 29, 32, 33] indicate the presence of auditory P3a in children and toddlers [34]. This difference between the visual and auditory modalities would be attributed to the possibility that some P3a generators in the auditory modality situated outside the frontal cortex would be able to contribute to frontal positivity during the P3a latency, while this contribution would not be as evident in the visual modality. The important contribution of the frontal cortex in generating the P3a has been obtained by means of neuropsychological [26, 27] and fMRI seeded dipoles [28]. Therefore, the absence of a clear P3a in the present study in children and adolescents can be attributed to the late maturation of the frontal cortex. Although young children, and even toddlers [34], present an auditory P3a, it has been argued that the auditory cortex is generating P3a, causing an inverted polarity at the mastoids, which is responsible for the P3a in the auditory modality [33, 35].

Our results confirm the presence of a visual P3b in children, measured as the difference between ERPs in target and standard conditions. The presence of a P3b with novel stimuli has been previously described by several authors, such as Courchesne [38]. The general maturational trend has been reported as a decrease in amplitude and latency with age [37, 52]. Furthermore, Flores et al. [62], in a central cue Posner paradigm, showed a decrease in P3b with age. The present results confirmed this reduction in P3b amplitude with age when the peak-to-peak amplitude of the P3b target modulation was considered. In addition, latency reduction with age was confirmed and inversely correlated with age. Sources of the P3b component are rather distributed, but they present a predominance of temporal and parietal sites. Main contributors to the P3b component would be located in the medial temporal lobe, *bilaterally* in the posterior parietal cortex, inferior parietal cortex and inferior temporal cortex [16, 28, 63]. The parietal sources were also confirmed by a distributed source model computed on individual brains [64]. The earlier maturation of posterior areas compared to frontal areas would not explain the differences between children and young adults in the amplitude and latency of the P3b component during the target condition. However, as indicated by Giedd et al. [40], the gradient of maturation is not only anterior-posterior, but also from low to high order association cortices, including the inferior parietal cortex, one of the neural generators of P3b.

The frontal SW (or LPN) presented a significant increase in the target condition compared to the standard condition. This component appears in frontal areas at a similar latency to P3b, and some authors [42, 65] have argued that in children it corresponds to the negative side of posterior positive dipoles. However, in the auditory modality, a genuine frontal origin for the SW has been demonstrated by a dissociation of the effects of the dorsolateral prefrontal cortex lesion on P3b and SW [27]. Dipole localization on the SW also indicated a frontal localization for this component [61]. The SW has been related to alerting and orienting [66]. This component is clearer in WM tasks as a response to the S1 stimulus [42, 44, 65], but it also appears in novelty and target detection tasks [27].

The lack of maturation of the frontal ERPs in children in the process of target selection of novel stimuli was demonstrated by the absence of P3a in children and pre-adolescents and by the age dependency of peak latency in the FSP component. Synaptic pruning and myelination of the frontal cortex continues to mature during the adolescent period [40, 67, 68]. The present results confirm the posterior-anterior gradient of brain maturation obtained with MRI techniques [40]. However, the posterior P3b component also showed an amplitude and latency maturation with age, indicating that a gradient of maturation from low order to high order association cortices also occurs in posterior areas [40]. As the children in the present experiment were highly competent in selecting targets, although there was certainly an improvement with age, the present results support the conclusion by Courchesne [38] using a similar experimental paradigm: “It is suggested that these differences in ERP waveforms reflect differences in the way children and adults categorize events.” The processes involved in target selection would be less represented in frontal areas in children, while they would progress to more anterior areas with age, completing this process around adolescence. The reorganization of brain locations for the implementation of cognitive functions during development has been recognized in Working Memory paradigms through experimental lesions in monkeys [69] and dipole localization of human ERPs [44], and it could be an additional basic developmental process.

It must be acknowledged that, although the overall sample size is large, the number of subjects for each year of age is somewhat small (i.e. 4 females and 4 males for each year of age). Furthermore, the present results correspond to a cohort study and not to a longitudinal study. Therefore, the generalization of the study results is reduced by these limitations.
